# Developing clinical skills assessment modules for traditional, complementary, and integrative medicine in Korea: a participatory action research study

**DOI:** 10.3352/jeehp.2026.23.10

**Published:** 2026-05-26

**Authors:** Yoonjin Jeong, Seung Hwan Mun, Eunbyul Cho, Hye-Yoon Lee, Sang Woo Shin, Soyeon Kim, Eui-hyoung Hwang, Man-suk Hwang, Eunseok Kim, Jungyun Lee

**Affiliations:** 1 School of Korean Medicine, Pusan National University, Yangsan, Korea; 2 Department of Diagnostics, College of Korean Medicine, Wonkwang University, Iksan, Korea; 3 Research Center of Traditional Korean Medicine, Wonkwang University, Iksan, Korea; 4 Division of Humanities and Social Medicine, School of Korean Medicine, Pusan National University, Yangsan, Korea; 5 Department of Applied Medicine, School of Korean Medicine, Pusan National University, Yangsan, Korea; 6 Department of Internal Medicine, Pusan National University Korean Medicine Hospital, Yangsan, Korea; 7 Department of Korean Medicine Rehabilitation, Spine and Joint Center, Pusan National University Korean Medicine Hospital, Yangsan, Korea; 8 Department of Acupuncture and Moxibustion Medicine, Pusan National University Korean Medicine Hospital, Yangsan, Korea; 9 Department of Sasang Constitutional Medicine, Pusan National University Korean Medicine Hospital, Yangsan, Korea; Hallym University, Korea

**Keywords:** Education, medical, Clinical competence, Educational measurement, Medicine, korean traditional, Acupuncture

## Abstract

**Purpose:**

This study aimed to develop pilot clinical skills assessment (CSA) modules for Korean medicine-specific procedures and to examine their preliminary appropriateness, perceived necessity, and feasibility as a foundation for future licensing-related assessment development.

**Methods:**

A participatory action research framework, supplemented by qualitative interviews, was used to develop 4 CSA modules—acupuncture, Chuna manual therapy, pulse diagnosis, and constitutional diagnosis—in collaboration with expert evaluators, students, and standardized patients. The modules were implemented as formative examinations for third-year Korean medicine students, after which semi-structured interviews were conducted to obtain feedback on module content, implementation processes, and scoring procedures. Each module was also reviewed using the RUMBA checklist (Realistic, Understandable, Measurable, Behavioral, and Achievable), together with ratings of perceived necessity and feasibility for possible future use in licensing-related assessment. Interview data were analyzed inductively at the level of individual responses and then compared across modules and participant groups.

**Results:**

Qualitative analysis yielded 3 themes: content and scoring criteria, physical environment or simulators, and education or training. Participants emphasized the need to make key aspects of performance more observable, improve authenticity through simulators or task trainers, and strengthen the capacity of scoring systems to distinguish between levels of student performance. Across all modules, mean RUMBA scores were high in the understandable, behavioral, and achievable domains, whereas measurability was more problematic, especially for pulse diagnosis.

**Conclusion:**

These pilot findings clarify both the strengths and the limitations of Korean medicine-specific CSA modules. The modules received favorable ratings for understandability and achievability, whereas lower ratings for measurability and realism identified priorities for refinement before wider use. This study provides preliminary guidance for the continued development and broader evaluation of Korean medicine-specific performance assessments.

## Graphical abstract

[Fig f5-jeehp-23-10]

## Introduction

### Background/rationale

Ensuring patient safety and treatment effectiveness in traditional, complementary, and integrative medicine (TCIM) requires systematic educational assessment of the clinical skills that directly shape procedural safety and quality of care. Assessment models developed for conventional medicine, however, cannot simply be transferred to TCIM when the target procedures involve profession-specific competencies, distinctive technical maneuvers, unique diagnostic frameworks, and distinct safety considerations [1,2].

Internationally, competency-based assessment frameworks and standardized clinical skills assessments (CSAs), including the objective structured clinical examination (OSCE), have been widely adopted in conventional medical education. By contrast, licensing-level assessment tools for TCIM procedures remain comparatively underdeveloped, despite the need for shared performance expectations and defensible scoring criteria [3]. In this study, CSA was used as the umbrella term for structured assessments of clinical performance, whereas OSCE referred specifically to the station-based format used in several of the proposed modules. Accordingly, the 4 modules were conceptualized as CSA modules, some of which adopted an OSCE-type structure.

Recent work in health professions education has emphasized that performance-based assessment requires not only relevant content, but also close attention to implementation feasibility and the defensibility of scoring decisions [4,5]. Reflecting this trend, East Asian countries with established TCIM systems have begun to introduce CSAs into licensing and training processes. China has implemented practical examinations for traditional Chinese medicine, including acupuncture and Tuina, and Taiwan has reported educational benefits from using OSCEs in traditional medicine training [3,6]. In South Korea, strategies for integrating CSAs into the Korean Medicine Doctor (KMD) licensing examination have likewise been explored, reflecting growing policy-level interest in standardized performance assessment [7].

### Objectives

Assessing these competencies poses substantial challenges related to observability, safety, authenticity, and discrimination between levels of learner performance in standardized examination settings [8]. Previous work in Korean medicine education has also identified ambiguities in scoring criteria and unresolved questions about the level of competence expected of undergraduates, underscoring the need for systematic module development and evaluation [8,9]. Therefore, this study aimed to develop and pilot CSA modules for 4 Korean medicine-specific clinical procedures using a participatory action research (PAR) framework and to examine their preliminary appropriateness, perceived necessity, and feasibility for future licensing-related assessment development [7].

## Methods

### Ethics statement

This study was approved by the Institutional Review Board (IRB) of Pusan National University (2023_90_HR). Written informed consent was waived by the IRB because the study retrospectively used anonymized data. Evaluators and developers participated as members of the PAR team.

### Personal characteristics of the research team

The research team comprised 6 professors: 3 men (S.W.S., M.S.H., and E.K.) and 3 women (H.Y.L., S.K., and J.L.). All team members held a Doctor of Korean Medicine degree and had experience conducting medical education research. Interviews were conducted by the fourth author (H.Y.L.), a female professor with a PhD (Doctor of Philosophy) in medical education and expertise in internal Korean medicine.

### Relationship with participants

Participants in the PAR framework included professors, students, and standardized patients (SPs). Because some members of the research team had previously taught the participating students, the existence of a prior professor-student relationship was explicitly acknowledged. Students were informed of the study objectives and were assured that participation would not affect their academic evaluation. The SPs had no prior relationship with the interviewer. Throughout the project, the research team reflected on potential sources of bias and incorporated stakeholder input through iterative review and revision meetings.

### Theoretical framework and methodology

This study was designed as a PAR-based pilot development and feasibility study and was conducted through 2 iterative cycles of planning, action, observation, and reflection. In the first cycle, 4 Korean medicine-specific procedures—acupuncture, Chuna manual therapy, pulse diagnosis, and constitutional diagnosis—were selected through document review, expert consultation, and previous policy-oriented work on CSA in Korean medicine as potential targets for future national-level CSA development. Based on recommendations from relevant academic societies regarding assessment items and methods [7], preliminary modules were created, including standardized case guidelines, SP scenarios, and checklist-based scoring tools (Table 1). In the second cycle, the modules were piloted with third-year Korean medicine students, and feedback was collected from evaluators, students, and SPs through questionnaires and semi-structured interviews. The modules were then revised through reflection and consensus meetings within the research team.

### Participant selection

Purposive sampling was used to include expert evaluators and SPs with relevant assessment expertise. Convenience sampling was applied to students who participated in clinical practice during the study period, because their formative assessment results were analyzed retrospectively. Eight expert evaluators were selected: 2 acupuncture specialists, 2 Korean rehabilitation medicine specialists, 1 Korean internal medicine specialist, 1 constitutional medicine specialist, and 2 medical education experts. Three students were assigned to the acupuncture module, and 3 different students were assigned to the Chuna manual therapy module. Two students participated in both the pulse diagnosis and constitutional diagnosis modules. Four SPs were designated, with one assigned to each module.

Given the developmental purpose of this pilot study, the sample was intended to capture information-rich feedback from key stakeholder groups rather than to achieve formal thematic saturation. Including evaluators, students, and SPs across the 4 modules was considered sufficient to identify major feasibility concerns and priorities for module refinement. No invited evaluators, students, or SPs declined to participate in the post-implementation feedback process, and no dropouts occurred after participation began.

### Setting and context

Educational activities and data collection were conducted during the second semester of 2022 at the School of Korean Medicine, Pusan National University, in the Republic of Korea. Ethical approval for the retrospective analysis was obtained in July 2023. Data analysis and manuscript preparation were completed by June 2025.

### Data collection

The modules were implemented as pilot formative CSAs for third-year Korean medicine students and were reviewed with standardized checklists by clinical content experts and medical education specialists. To promote standardization across modules, evaluators were oriented to the objectives of each station and reviewed the checklist items and scoring expectations before implementation. SPs received structured preparation regarding case flow, expected responses, and safety precautions. For procedure-based modules, written instructions and demonstration-based guidance were provided to reduce variability in task delivery. Evaluators were instructed to apply the scoring criteria consistently according to the predefined assessment framework.

After the examinations, expert evaluators, students, and SPs completed paper-based questionnaires and participated in semi-structured interviews. The authors developed the interview questions based on the RUMBA checklist—Realistic, Understandable, Measurable, Behavioral, and Achievable—and on comparisons with existing OSCE examinations. In the RUMBA checklist, “Realistic” refers to practical feasibility, “Understandable” to clarity for learners, “Measurable” to objective assessment, “Behavioral” to observable actions, and “Achievable” to attainability by learners [10,11]. Interviews were documented through contemporaneous note-taking rather than audio recording to protect anonymity. Data were recorded in real time using a laptop and structured worksheets. Interviews lasted approximately 1 hour per case. Repeat interviews were not conducted; each participant contributed one post-implementation feedback session for the modules in which they were involved.

### Data analysis

Two researchers (H.Y.L. and E.C.) independently reviewed the field notes and generated initial codes using an inductive approach, with the individual interview response serving as the primary unit of analysis. Codes were first generated at the response level and then compared across modules and participant groups to identify both cross-cutting and module-specific themes. The codes were subsequently grouped into categories and synthesized into broader themes through iterative discussion (Dataset 1).

To enhance credibility, discrepancies were resolved through consensus, and the thematic structure was reviewed by the research team. The final coding framework comprised 3 overarching themes: content and scoring criteria, physical environment or simulators, and education or training. Detailed categories are presented in Supplement 1.

In addition, each module was reviewed using the RUMBA checklist, together with 5-point Likert ratings of perceived necessity and feasibility for possible future use in licensing-related assessment (Dataset 2). The RUMBA checklist was used as a practical review framework rather than as a source of validation evidence. The modules were subsequently revised to improve authenticity, safety, and implementation feasibility.

## Results

### Stakeholder feedback from interviews

The qualitative responses for each module are presented in Tables 2 and 3. Evaluators’ suggestions for module improvement were organized into 3 overarching themes: content and scoring criteria, physical environment or simulators, and education or training. These themes captured concerns that recurred across modules, as well as module-specific issues related to observability, authenticity, safety, and discrimination between levels of student performance.

In the acupuncture OSCE, feedback emphasized the need to strengthen assessment of landmark-based needling safety and expand evaluation of manipulation techniques. In the Chuna manual therapy OSCE, accurate grading was limited by poor visibility of hand placement, leading evaluators to recommend close-up camera or monitoring systems. In the pulse diagnosis OSCE, evaluators suggested broadening the assessment to include multiple pulse patterns and modifying the station setup to improve visibility. In the constitutional diagnosis OSCE, evaluators recommended clearer specification of differential diagnoses and a higher level of module difficulty.

Ten responses were collected from 8 students, including 2 students who participated in 2 modules, and 4 responses were collected from the 4 SPs. Their feedback focused primarily on authenticity and safety. Suggested improvements included using auxiliary equipment and developing assessment methods that could more effectively distinguish between levels of student performance.

### RUMBA checklist and necessity & feasibility scores

Using the RUMBA checklist, the 4 modules received mean scores of 4 or higher in the understandable, behavioral, and achievable domains. The acupuncture module received scores of 4 or higher across all RUMBA domains.

**Fig. 1.**
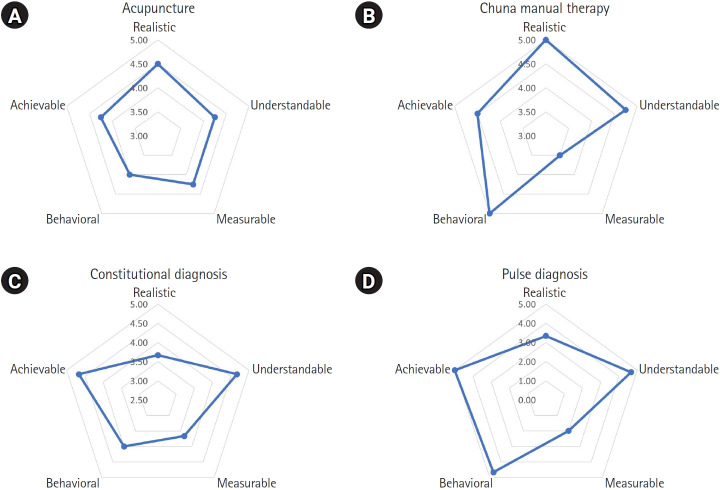
RUMBA checklist (Realistic, Understandable, Measurable, Behavioral, and Achievable) ratings across the 4 modules. (A) Acupuncture. (B) Chuna manual therapy. (C) Constitutional diagnosis. (D) Pulse diagnosis.

Across modules, understandability, behavioral alignment, and achievability were consistently rated highly, whereas measurability was the most challenging domain. Measurability was the lowest-rated domain for Chuna manual therapy (3.5), constitutional diagnosis (3.67), and pulse diagnosis (2), and constitutional diagnosis also showed a comparatively lower score for realism (3.67) (Fig. 1).

**Fig. 2.**
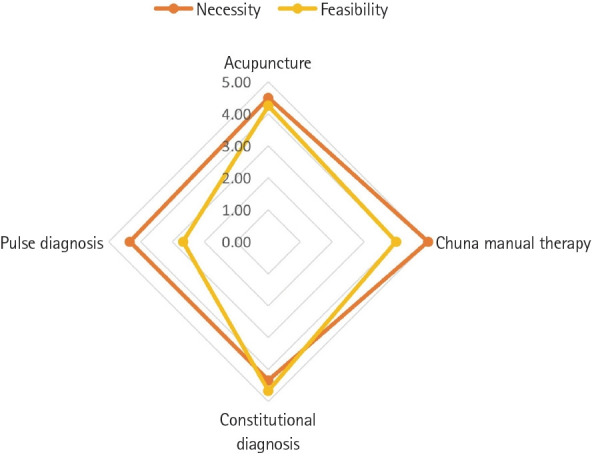
Perceived necessity and feasibility ratings across the 4 modules.

Chuna manual therapy had the highest perceived necessity score (mean, 5.0), followed by acupuncture (4.5), pulse diagnosis, and constitutional diagnosis (both 4.33). By contrast, perceived feasibility was highest for constitutional diagnosis (4.67) and lowest for pulse diagnosis (2.67), indicating a discrepancy between perceived necessity and feasibility across modules (Fig. 2).

### Module refinements informed by pilot feedback

**Fig. 3.**
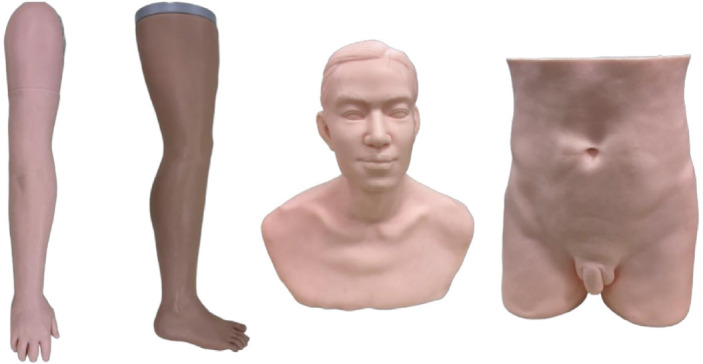
Arm, leg, head, and torso mannequins used for acupuncture skills.

**Fig. 4.**
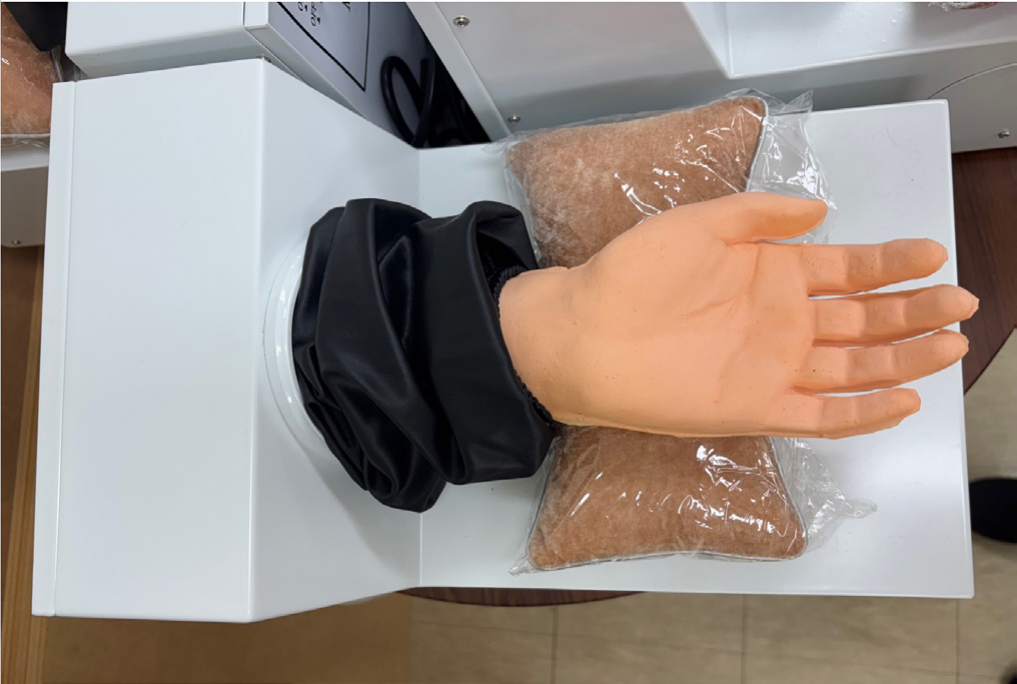
Pulse simulator used for pulse diagnosis skills.

The modules were refined based on feedback addressing observability, authenticity, and standardization. Regional anatomical mannequins were introduced to improve assessment accuracy and standardization in the acupuncture module (Fig. 3). For Chuna manual therapy, additional mannequin-based approaches were explored to improve observation of cervical techniques. The pulse diagnosis module was restructured from a procedural task into a clinical encounter OSCE format that incorporated a pulse simulator (Fig. 4). For constitutional diagnosis, the SP pool was expanded in collaboration with specialists to better support constitution-specific assessment.

## Discussion

### Key results

This pilot study developed and tested 4 Korean medicine-specific CSA modules through a PAR process. Feedback from evaluators, students, and SPs identified recurring problems involving observability, authenticity, safety, and discriminatory scoring. Quantitative ratings showed high levels of understandability and achievability across modules, whereas measurability remained difficult, particularly for pulse diagnosis. Taken together, these findings identify practical priorities for refinement before broader implementation [12,13].

### Interpretation

#### Acupuncture

The acupuncture module received favorable ratings across the RUMBA domains, suggesting that it may be feasible for future licensing-related CSA development. Interpreting these findings, however, requires a clear distinction between the clinical acupuncture competence that should be assessed at the national licensure level and the knowledge of acupuncture point theory typically taught during undergraduate education [8].

At the licensing level, acupuncture should be assessed as a clinical procedure that emphasizes patient safety and procedural judgment, rather than as an isolated test of meridian knowledge or point localization. Directly assessing acupoint selection in time-limited OSCEs may introduce construct-irrelevant variance. Therefore, point selection may be more appropriately evaluated through written or case-based examinations, whereas CSAs may be better suited to assessing the safe execution of needling.

Overall, licensing-level acupuncture CSAs should prioritize procedural safety, patient-centered interaction, and infection control, while selectively incorporating technical elements that are directly related to risk prevention. This approach would support clinically meaningful and profession-specific assessment standards for KMDs [7].

#### Chuna manual therapy

For Chuna manual therapy, standardization emerged as the central challenge for high-stakes assessment, because identical techniques would need to be applied repeatedly to ensure fairness across examinees. In this context, exclusive reliance on SPs raises safety concerns, as repeated manual manipulation may pose cumulative physical risks and introduce variability related to individual body characteristics.

Accordingly, CSA modules for Chuna manual therapy should prioritize assessment models that protect both standardization and safety. Mannequins or task trainers represent practical alternatives for high-stakes examinations because they allow repeated application of techniques without compromising participant safety. Evaluators also emphasized the need for objective verification of performance, particularly with respect to hand placement, contact points, and movement trajectories. Supplementary evaluation tools, such as proximity cameras, video recording, or sensor-based devices, may therefore improve observability and scoring transparency.

These findings suggest that licensing-level CSAs for Chuna manual therapy should be supported by hybrid assessment environments that combine safe physical models with objective observation technologies [14-16]. Such an approach is consistent with the goals of patient safety and defensible, standardized evaluation of profession-specific manual skills [17,18].

#### Pulse diagnosis

The findings revealed 2 critical limitations of the current pulse diagnosis module. First, reproducing diverse pathological pulse conditions with SPs alone was difficult in a standardized examination format, limiting the assessment of diagnostic accuracy. Second, pulse diagnosis is usually performed during patient-physician consultations at a desk, which makes close observation by evaluators challenging. Determining which competencies should be assessed, and how they should be operationalized, therefore remains unresolved, particularly in the absence of routinely implemented simulators.

Whether the examination should evaluate procedural steps alone or also assess the ability to diagnose pathological pulse conditions accurately remains controversial. Expert feedback in this study supported assessing not only the procedure itself, but also the ability to interpret and diagnose pathological pulse conditions. Careful consideration is therefore needed when defining competency levels for undergraduate education and licensing examinations.

#### Constitutional diagnosis

Two challenges complicate the integration of constitutional diagnosis into CSAs. First, constitutional medicine incorporates patients’ body type and appearance, requiring SPs who are appropriately matched to the intended constitution. Second, actual constitutional diagnosis typically requires more than 12 minutes and additional questionnaires [19], making it difficult to incorporate into a conventional OSCE format.

Measurability was also low because assessing only post-diagnostic care based on a provided constitution made it difficult to distinguish between levels of student performance. The objective of the examination was not limited to identifying the constitution itself; rather, it also involved presenting a disease diagnosis related to the constitution. Thus, the OSCE was designed to assess competencies such as history taking, physical examination, patient education, and patient-physician interaction.

Future studies should validate OSCE designs that integrate constitution identification, diagnostic reasoning, and management within feasible time limits, using SPs who reflect typical constitutional characteristics. Although an SP pool was developed in this study, nationwide examinations would require region-specific SP pools that account for the availability of SPs.

#### Broader validity framework

The present findings should not be interpreted as evidence from a full validation study. Rather, the RUMBA ratings and stakeholder feedback provide preliminary evidence regarding content appropriateness, feasibility, and priorities for refinement. Broader validation of Korean medicine-specific CSA modules will require additional evidence, including reliability, response process documentation, relationships with other measures, and assessment consequences in larger multi-institutional studies [14].

### Limitations

This study has several limitations. First, it was conducted at a single institution with a small pilot sample, which limits the transferability of the findings. Second, because this was a developmental pilot study, it was not designed to evaluate inter-rater reliability or other psychometric properties. Third, interviews were documented through contemporaneous note-taking rather than audio-recorded transcription, which may have limited the capture of nuanced responses, although real-time documentation was used to support consistency. Fourth, although the modules were developed with licensing relevance in mind, the findings should be interpreted as preliminary and developmental rather than as evidence supporting immediate high-stakes implementation.

### Generalizability/suggestions

This study provides situational guidelines and examination room preparation instructions in Supplements 2 and 3 to support reproducibility for traditional medicine educators. Unlike previous OSCE studies that used self-developed surveys to investigate examinee satisfaction [8,9,20], this study used the RUMBA checklist to assess the appropriateness of the 4 simulation-based modules and identify areas for improvement. Diverse feedback from evaluators was essential for modifying and refining the CSA modules. On the basis of the findings of this pilot study, multi-institutional research with larger numbers of examinees is needed to examine reliability, response processes, feasibility at scale, and broader validity evidence for Korean medicine-specific CSA modules [21].

### Conclusion

Through the application of a PAR process, this study developed and piloted 4 Korean medicine-specific CSA modules that incorporated input from students, medical education experts, clinical experts, and SPs. The findings suggest that, although the modules received favorable ratings for understandability and achievability, lower ratings for measurability and realism highlighted areas requiring further refinement. Specific priorities include the development of specialized simulators or task trainers, improved observation systems, and more structured SP preparation and evaluator calibration. These findings provide preliminary guidance for refining Korean medicine-specific performance assessments and for designing future larger-scale studies before these assessments are considered for high-stakes licensing contexts.

## Figures and Tables

**Figure f5-jeehp-23-10:**
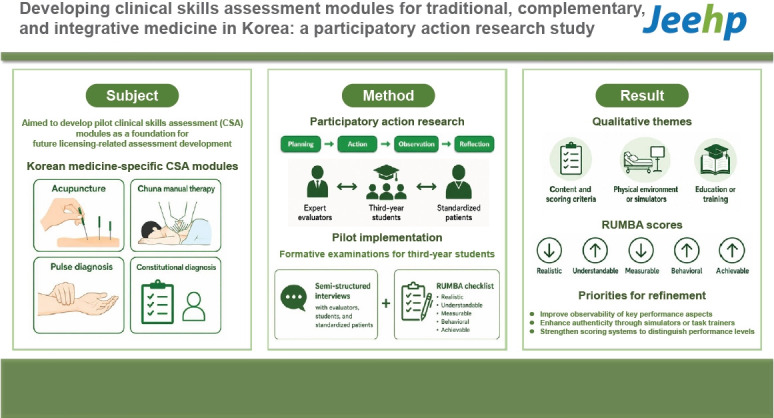


**Table 1.** Proposed criteria and procedures for assessing 4 clinical skills in integrative medicine

Module	Subject	Assessment content	Procedures
Acupuncture (OSCE)	SP and a model (acupuncture training pad)	Hand hygiene, patient instruction, point location, needle insertion depth and direction, manipulation technique (lifting/thrusting and twirling), needle removal, and patient guidance	Explain the procedure to the SP, position the patient, perform hand hygiene, mark acupoints with stickers, educate the SP, insert, manipulate, and withdraw the needle on a training pad, monitor the SP for adverse events, and explain post-procedure precautions
Chuna manual therapy (OSCE)	SP	Hand hygiene, guidance on patient posture, and Chuna technique, including joint mobilization, joint distraction, and muscle energy technique	Perform the specified Chuna manual technique, using low-risk manipulation, on the SP and provide instructions by explicitly stating the name of the Chuna technique and the treatment site, such as the left or right side
Pulse diagnosis (OSCE)	SP	Patient instructions, guidance on patient posture, pulse location, and diagnostic results	Conduct pulse diagnosis on the SP and explain findings based on pulse speed within 5 minutes
Constitutional diagnosis (OSCE)	SP	History taking, physical examination, patient education, and planning based on a predefined constitution	Conduct a clinical consultation using SP scenario guidelines, including history taking and physical examination, explain the diagnostic reasoning, and provide patient education tailored to the specified constitution

OSCE, objective structured clinical examination; SP, standardized patient.

**Table 2.** Evaluators’ responses on each module

Module	Content/scoring criteria	Physical environment/simulators	Education/training
Acupuncture (OSCE)	“Sticker-marking omits most bone-based proportional measurement and point palpation, and the checklist does not require those actions. Insertion depth is hard to judge when needling the pad.” (P1)	“It would be more realistic if the acupuncture pad were placed on the side rather than under the patient’s foot.” (P1)	“Students are uncertain about the required actions, so clearer instruction is needed.” (P4)
	“Increase the points allocated to acupoint identification.” (P2)	“Replace the clinic wall clock with a silent model. Provide 2 sets of arrow stickers, distinguishing reinforcing from reducing, in blue/red or black/white.” (P3)	
	“Acupuncture needling of the Eight Confluence Points should assess rotation, lifting-thrusting, and reinforcing-reducing techniques.” (P3)	“Configure the station for greater authenticity, such as by using a wearable acupuncture pad.” (P4)	
Chuna manual therapy (OSCE)	“For the cervical JS technique, the contact point is invisible; consider removing this item.” (P6)	“To protect the SP, developing a model that makes contact with the transverse process or articular pillar visible is crucial.” (P5)	“Detailed pre-examination instruction is needed.” (P5)
	“Fine anatomical landmarks, such as the cervical transverse process and articular pillar, cannot be visualized, hindering accurate scoring. In shoulder-joint techniques, discriminating performance differences among students is difficult.” (P1)	“Judging whether the maneuver moves from C6 up to C2 requires a close-up camera-monitor system.” (P1)	
		“Because the transverse and spinous processes are difficult to visualize, mannequins are needed.” (P4)	
Pulse diagnosis (OSCE)	“Assessing only slow vs. rapid pulse is too easy; the test should also require identification of wiry, strong, weak, slippery, and other pulse patterns.” (P4)	-	-
	“The examinee’s interpretation of the pulse findings should also be evaluated.” (P1)		
	“Whether the candidate is palpating the Cun-Guan-Chi positions correctly is difficult to observe clearly.” (P7)		
Constitutional diagnosis (OSCE)	“The scoring sheet and student training both need a sharper focus. Broaden and clarify the disease categories to be differentiated and the questions used to distinguish constitutional patterns.” (P1)	-	“Examinees show a general lack of knowledge about the differential diagnosis of tremor; additional disease-diagnosis training is required. Examinees’ understanding of the OSCE format itself is insufficient.” (P8)
	“Increase the point allocation for constitutional-disease items; the current case is too easy.” (P4)		

Expert evaluators’ responses are coded as P1–P8. OSCE, objective structured clinical examination; SP, standardized patient.

**Table 3.** Students’ and standardized patients’ opinions on each module

Module	Students	SPs
Acupuncture (OSCE)	“Attaching stickers is not authentic.” (S1)	“Removing stickers completely within the limited break time was challenging. Sticker adhesiveness was reduced by body hair and sweat.” (SP1)
	“Attaching stickers to the SP and inserting an acupuncture needle into a model are awkward, and understanding the direction is difficult.” (S2)	
	“Inserting and removing needles on a model, checking for side effects, and cautioning SPs are awkward.” (S3)	
Chuna manual therapy (OSCE)	“I was worried that the professors might not clearly see the location of the test takers’ hands and fingers.” (S4)	“Neck and head pain. I was worried that the manipulation was too forceful.” (SP2)
	“I tended to use more power than usual because I was nervous and worried that the maneuver would not be judged sufficient.” (S5)	
	“More training and preparation for the clinical skills examination are needed.” (S6)	
Pulse diagnosis (OSCE)	“I expected a normal pulse because SPs, rather than mannequins, were used.” (S7)	“Pressure differed depending on the test taker.” (SP3)
	“I was concerned that evaluators might not clearly see finger placement.” (S8)	
Constitutional medicine (OSCE)	“Providing the constitution in advance is unrealistic.” (S7)	“The focus on overall health status was notable. It was not difficult to respond as a standardized patient.” (SP4)
	“There are too many items to be evaluated.” (S8)	

Students’ responses are coded as S1–S8; standardized patients’ responses are coded as SP1–SP4. OSCE, objective structured clinical examination; SP, standardized patient.

## Footnotes

**Data availability**
Data files are available from https://doi.org/10.7910/DVN/4ZWMQB Dataset 1. Interview field notes.

Dataset 2. RUMBA checklist and necessity and feasibility scores.

## Supplementary materials

Supplement 1. Coding framework for qualitative analysis.

Supplement 2. Pilot modules of clinical skills assessments developed in this study.

Supplement 3. Pre-examination guidance for examinees, standardized patients, and scorers of the acupuncture OSCE.

Supplement 4. Audio recording of the abstract.
