# Supplementary material for: Developing clinical skills assessment modules for traditional, complementary, and integrative medicine in Korea: a participatory action research study
Source: J Educ Eval Health Prof. 2026 May 26;23:10. doi: 10.3352/jeehp.2026.23.10 (PMC13311766; doi:10.3352/jeehp.2026.23.10)
Supplement: Supplementary file 3 — Supplement 1. Coding framework for qualitative analysis. [file jeehp-23-10-suppl1.docx]

**Supplement 1.** Coding framework for qualitative analysis

| Overarching theme | Category | Description |
| --- | --- | --- |
| Content and scoring criteria | Scope and adequacy of assessment content | Comments addressing whether the module covered sufficiently important procedures, diagnostic elements, or clinical tasks |
|  | Clarity and appropriateness of scoring criteria | Comments addressing checklist structure, point allocation, and the appropriateness of scoring items |
|  | Discrimination between performance levels | Comments addressing whether the module could distinguish differences in student performance |
| Physical environment or simulators | Observability of performance | Comments addressing whether evaluators could clearly observe hands, fingers, contact points, or procedural steps |
|  | Authenticity and realism of the assessment setting | Comments addressing how closely the station reflected actual clinical practice |
|  | Need for simulators, mannequins, or equipment support | Comments recommending devices or setup changes to improve realism, safety, or scoring |
|  | Safety concerns during performance | Comments addressing physical risk or discomfort during repeated procedures |
| Education or training | Examinee preparation and familiarity | Comments addressing students’ need for clearer instructions, more preparation, or greater familiarity with the OSCE format |
|  | Knowledge and skill readiness | Comments addressing the need for stronger prior learning or skill development before assessment |
|  | Standardization of evaluator/SP preparation | Comments implying the need for clearer alignment in administration, responses, or scoring |

OSCE, objective structured clinical examination; SP, standardized patient.
