# Supplementary material for: Developing clinical skills assessment modules for traditional, complementary, and integrative medicine in Korea: a participatory action research study
Source: J Educ Eval Health Prof. 2026 May 26;23:10. doi: 10.3352/jeehp.2026.23.10 (PMC13311766; doi:10.3352/jeehp.2026.23.10)
Supplement: Supplementary file 4 — Supplement 2. Pilot modules of clinical skills assessments developed in this study. [file jeehp-23-10-suppl2.docx]

**Supplement 2.** Pilot modules of clinical skills assessment developed in this study

**1. Acupuncture**

| **1) Station setting** |
| --- |
| • The examination is conducted in a room equipped with a consultation desk, chairs, and an examination coach.  • Place an acupuncture-training pad on a cart beside the couch.  • Two examiners remain inside the room; 2 additional examiners observe through a one-way mirror outside the room. |
| **2) Scenario instructions** |
| Item 1. Acupuncture at the Seven Stroke-specific Points (GV20, GB7, GB21, LI11, GB31, ST36, GB39) |
| [Instructions outside the examination room—to be read 1 minute before entry]  (1) Palpate the patient to locate all 7 stroke-specific points; attach a sticker so that its center lies directly over each acupoint. For bilateral points, mark only one side.  (2) Following the in-room instructions, perform needling on the acupuncture training pad.  [Instructions inside the examination room]  Assume the training pad represents a human body and perform acupuncture needling according to the following instructions.  • Point: GV20  • Needle direction: along the course of the meridian  • Angle: transverse insertion  • Depth: 1 cm  • Technique: After insertion, perform lifting–thrusting manipulation for 5 seconds to elicit de-qi, then withdraw the needle. |
| Item 2. Acupuncture at the Liver-Tonification Prescription (Sa-am Acupuncture) (Tonify KI 10 & LR 8; Sedate LU 8 & LR 4) |
| [Instructions outside the examination room—to be read 1 minute before entry]  (1) Palpate the constitutional points of the Liver-tonification prescription; affix an arrow so that its tip indicates the point and its shaft shows the needle direction according to the Yeong-Sui supplementation–drainage method. Mark one side only.  (2) Following the in-room instructions, perform needling on the acupuncture pad.  [Instructions inside the examination room]  Assume the training pad represents a human body and perform acupuncture needling according to the following instructions.  • Point: LU8  • Needle direction: per the Yeong-Sui supplementation–drainage method  • Angle: oblique insertion  • Depth: 0.5 cm  • Technique: Confirm de-qi and immediately withdraw the needle. |
| Item 3. Acupuncture at the Eight Confluence Points |
| [Instructions outside the examination room—to be read 1 minute before entry]  (1) Palpate and mark the Eight Confluence Points on one side of the body, centering a sticker over each point.  (2) Following the in-room instructions, perform filiform needling on the acupuncture pad.  [Instructions inside the examination room]  Assume the training pad represents a human body and perform acupuncture needling according to the following instructions.  • Point: ST36  • Angle: Perpendicular insertion  • Depth: 1 cm  • Technique: After insertion, twirl the needle for 5 seconds to elicit de-qi, then withdraw the needle. |

**2. Chuna manual therapy**

| **1) Station setting** |
| --- |
| • The examination is conducted in a room equipped with a consultation desk, chairs, and a Chuna therapy table.  • Two examiners assess inside the room; 2 others observe via a one-way mirror outside the room. |
| **2) Scenario instructions** |
| Item 1. Side-Lying Scapulothoracic Joint Mobilization |
| Assuming the standardized patient has a right-shoulder problem, perform side-lying scapulothoracic joint mobilization up to 5 times. |
| Item 2. Cervical JS Distraction Manipulation |
| Perform the cervical JS distraction manipulation technique twice for each cervical segment on the standardized patient. |

3. Pulse diagnosis

| 1) Station setting |
| --- |
| - The examination is conducted in a room equipped with a consultation desk, chairs, and a digital clock displaying seconds on the desk. |
| 2) Scenario instructions |
| Conduct the correct pulse-diagnosis procedure, assess the right Cun, Guan and Chi pulses, determine whether each pulse is slow, normal or rapid, and explain your findings to the patient |

4. Constitutional diagnosis

| **1) Station setting** |
| --- |
| - The examination is conducted in a room equipped with a consultation desk, chairs, and an examination coach. - Three examiners score inside the room; the standardized patient scores the patient–physician interaction (PPI) domain. |
| **2) Scenario instructions** |
| A 42-year-old female of Tae-eum constitution, presents with hand tremor. Vital signs are provided. Take a symptom-focused history, perform an appropriate physical examination, then discuss your provisional diagnosis and further plans for investigation and treatment with the patient. |
