# Supplementary material for: Developing clinical skills assessment modules for traditional, complementary, and integrative medicine in Korea: a participatory action research study
Source: J Educ Eval Health Prof. 2026 May 26;23:10. doi: 10.3352/jeehp.2026.23.10 (PMC13311766; doi:10.3352/jeehp.2026.23.10)
Supplement: Supplementary file 5 — Supplement 3. Pre-examination guidance for examinees, standardized patients, and scorers of the acupuncture OSCE. [file jeehp-23-10-suppl3.docx]

**Supplement 3.** Pre-exam guidance for examinees, standardized patients, and scorers for the acupuncture OSCE

**1. Guidance for examinees**

• Trim and clean nails before the exam.

• After entering the exam room, check the instruction, and the exam begins when the evaluator sets the time limit.

• For the patient, only identify point location; do not perform acupuncture. Acupuncture should only be done on the designated training pad.

• Disinfection is not required during acupoint exploration.

• The use of an acupuncture tube is irrelevant to the evaluation and can be decided by the examinee.

• If the examinee is pricked by a needle during the procedure, immediately disinfect the wound, confirm hemostasis, and apply a bandage. Then, continue the exam. Depending on the examinee’s condition, the evaluator may arbitrarily stop the exam and prioritize appropriate treatment.

• Stop actions immediately when the time limit expires.

• If completed before the time limit, please notify the evaluator that you have completed it.

• Upon exam completion, organize/dispose of used items and exit.

• Respect the patient and do not perform actions harmful to the patient.

• Do not engage in conversations unrelated to the exam with the patient.

• Do not communicate with the evaluator during the examination.

**2. Guidance for standardized patients**

• Sit on the couch at the start of the exam.

• Do not perform actions not guided by the examinee.

• Do not engage in conversations unrelated to the exam with the examinee.

• Do not communicate with the evaluator during the assessment.

• If the examinee asks for consent before the procedure, respond with “yes.”

• If the examinee guides the posture before the procedure, take the position accordingly.

• If the examinee asks about sensations during the procedure (such as soreness, tingling, pulling, heaviness, itching, comfort, coolness, warmth, etc.), respond with “yes.”

• If the examinee asks about possible adverse reactions after the procedure (such as pain or sensory abnormalities), respond with “no.”

**3. Guidance for scorers**

• Verify the examinee’s identification information (name, examination number).

• Review the scoring criteria beforehand and closely observe the examinee’s actions while scoring.

• Circle the scores for each item during scoring.

• If the evaluation criteria seem ambiguous during the assessment, record specifically.

• If the examinee is pricked by a needle during the procedure, immediately disinfect the wound, confirm hemostasis, and apply a bandage. Then, continue the exam. If bleeding continues or the exam becomes difficult, the evaluator may arbitrarily stop the exam and prioritize appropriate treatment.

• If the time limit expires, record all unperformed items as 0 points.

• Do not communicate with the examinee or standardized patient during the examination.
